# Partial Alleviation of Homologous Superinfection Exclusion of SeMNPV Latently Infected Cells by G1 Phase Infection and G2/M Phase Arrest

**DOI:** 10.3390/v16050736

**Published:** 2024-05-06

**Authors:** Qi-Ming Fu, Zheng Fang, Lou Ren, Qing-Shan Wu, Jun-Bo Zhang, Qiu-Ping Liu, Lei-Tao Tan, Qing-Bei Weng

**Affiliations:** 1 School of Life Sciences, Guizhou Normal University, Guiyang 550025, China; fuqiming95@163.com (Q.-M.F.); zhengfang03@126.com (Z.F.); renlou0512@163.com (L.R.); wqs288@126.com (Q.-S.W.); z5507737@163.com (J.-B.Z.); liuqiuping0999@163.com (Q.-P.L.); little.tan@163.com (L.-T.T.); 2 College of Biological Science and Agriculture, Qiannan Normal University for Nationalities, Duyun 558000, China

**Keywords:** cell cycle, SeMNPV, *Spodoptera exigua*, latent infection, superinfection exclusion, cell cycle arrest

## Abstract

Viral infection can regulate the cell cycle, thereby promoting viral replication. Hijacking and altering the cell cycle are important for the virus to establish and maintain a latent infection. Previously, Spodoptera exigua multiple nucleopolyhedrovirus (SeMNPV)-latently infected P8-Se301-C1 cells, which grew more slowly than Se301 cells and interfered with homologous SeMNNPV superinfection, were established. However, the effects of latent and superinfection with baculoviruses on cell cycle progression remain unknown. In this study, the cell cycle profiles of P8-Se301-C1 cells and SeMNPV or Autographa californica multiple nucleopolyhedrovirus (AcMNPV)-infected P8-Se301-C1 cells were characterized by flow cytometry. The results showed that replication-related genes *MCM4*, *PCNA*, and *BAF* were down-regulated (*p* < 0.05) in P8-Se301-C1 cells, and the S phase of P8-Se301-C1 cells was longer than that of Se301 cells. P8-Se301-C1 cells infected with SeMNPV did not arrest in the G2/M phase or affect the expression of *Cyclin B* and cyclin-dependent kinase 1 (*CDK1*). Furthermore, when P8-Se301-C1 cells were infected with SeMNPV after synchronized treatment with hydroxyurea and nocodazole, light microscopy and qRT-PCR analysis showed that, compared with unsynchronized cells and S and G2/M phase cells, SeMNPV-infected P8-Se301-C1 cells in G1 phase induced G2/M phase arrest, and the amount of virus adsorption and intracellular viral DNA replication were significantly increased (*p* < 0.05). In addition, budded virus (BV) production and occlusion body (OB)-containing cells were both increased at 120 h post-infection (*p* < 0.05). The expression of *Cyclin B* and *CDK1* was significantly down-regulated at 48 h post-infection (*p* < 0.05). Finally, the arrest of SeMNPV-infected G1 phase cells in the G2/M phase increased BV production (*p* < 0.05) and the number of OB-containing cells. In conclusion, G1 phase infection and G2/M arrest are favorable to SeMNPV proliferation in P8-Se301-C1 cells, thereby alleviating the homologous superinfection exclusion. The results contribute to a better understanding of the relationship between baculoviruses and insect cell cycle progression and regulation.

## 1. Introduction

The cell cycle is a rhythmic process of cell proliferation that plays a crucial role in maintaining the normal growth and division of the cell [1]. The eukaryotic cell cycle is generally divided into four phases: DNA synthesis phase (S), mitotic phase (M), and two gap phases (G1 and G2), and progression through each phase is tightly regulated and highly orchestrated in the cellular processes. Cell cycle progression in all eukaryotes is controlled by an intricate mechanism involving cyclins and cyclin-dependent kinases (CDKs), as well as other factors [2]. As part of their pathogenesis, many viruses are known to have evolved multiple strategies to manipulate host cell cycle progression by regulating cyclin and CDK expression [3,4], which may inhibit the early death of infected cells, thereby allowing cells to escape from immune defenses and promoting viral assembly [5,6]. Viral infection induces cell cycle arrest in G1, S, or G2/M phases to exploit the host cell synthesis machinery, utilize cellular DNA replication material, utilize cytoskeletal transport, or evade innate immune sensing [4,7,8,9,10,11,12].

Baculoviruses are rod-shaped nucleocapsid viruses with circular double-stranded DNA genomes. They are specific pathogens of insects, especially Lepidoptera insects [13]. The life cycle of a canonical baculovirus has two divergent virion morphologies, including occlusion-derived virus (ODV) and budded virus (BV). ODVs are occluded by a protein matrix, forming occlusion bodies (OBs). Upon ingestion of food contaminated with OBs, ODVs are released by the midgut’s alkaline pH, initiating a primary infection by infecting epithelial cells of the midgut. The BVs are produced from the infected midgut epithelial cells and disperse to infect other cells during the systemic phase of infection [14]. There is increasing evidence that baculovirus infection actively manipulates cell cycle progression to provide favorable conditions for its own replication. Autographa californica multiple nucleopolyhedrovirus (AcMNPV) [15] and *Bombyx mori* nucleopolyhedrovirus (BmNPV) [16] infection elicit cell cycle arrest in S or G2/M phase via multiple viral genes or viral protein expression, such as immediate early gene *ie2* [17], late expression factor gene *lef-11* [11], structural protein gene *ODV-EC27* [18], and inhibitors of apoptosis proteins (*IAPs*) [16]. In cultured insect cells, AcMNPV can establish infection in cells at different cell cycle phases and induce cell cycle arrest in the S phase or G2/M phase, but infecting G1 or S phase cells can arrest cells in the S phase more rapidly [19]. In addition, AcMNPV infection of G1 phase cells can yield a higher number of progeny viruses (BV) than in other phases [20]. Thus, different phases of the cell cycle exhibit varying susceptibilities to viral infection.

Baculoviruses are highly pathogenic and can cause epidemics of viral diseases; they are being used as bioinsecticides for pest control [21]. In some cases, however, baculovirus infection does not necessarily exhibit an acute infection leading to insect death but instead establishes a covert infection with no apparent symptoms [22]. Covert infections of baculoviruses have been reported to be prevalent in insect populations in the field [23,24,25], which can severely limit the application range and insecticidal efficiency of baculovirus insecticides. Covert infection includes persistent and latent infections. During persistent infections, some viral genes are downregulated, and a small number of daughter viruses can continue to be produced [26]. A latent infection is defined as a reversible, nonproductive infection in which infectious viruses are not produced. During viral latency, although no infectious virus can be detected, the viral genome is present in the host cells, with very few or no gene expressions present [27,28]. It has been reported that Bovine herpesvirus 1 (BHV-1), Epstein–Barr virus (EBV), and Kaposi’s sarcoma-associated herpesvirus (KSHV) express certain viral latency-related genes, such as latent membrane proteins and latency-associated nuclear antigens, which can regulate the host cell cycle progression, induce G1 phase arrest, or promote G1/S transition, leading to changes in cell proliferation rate and abnormal cell proliferation of latently infected cells [29,30,31,32]. Therefore, hijacking and altering the cell cycle are important for the virus to establish a latent infection. However, little is known about the effect of baculovirus latent infection on cell cycle progression in insect hosts.

Latently infected cells often develop resistance to subsequent infections with the same or homologous viruses (a phenomenon also known as superinfection exclusion, SIE) [26]. Neither gene expression nor genome replication of the superinfecting virus occurred [33]. Superinfection exclusion may occur at different infection stages after virus infection, such as membrane fusion, virion attachment, invasion, and DNA replication [33,34,35], and seems to be a prerequisite for the maintenance of viral latency [36]. Beperet et al. found that the homologous superinfection exclusion depends on the time interval between infections, and the instantaneous window within the interval allowed for specific heterospecific alpha-baculovirus superinfection [37]. The interaction between latently infected virus and cells and the mechanism of superinfection exclusion are complicated, and the effect of baculovirus latent infection on insect cell cycle progression needs to be further studied.

Previously, Spodoptera exigua multiple nucleopolyhedrovirus (SeMNPV)-latently infected P8-Se301-C1 cells were established by infecting *Spodoptera exigua* Se301 cells with undiluted passaged SeMNPV [26]. P8-Se301-C1 cells harbored a partial SeMNPV genome and some SeMNPV transcripts. The cells showed a slower growth rate than Se301 cells and displayed inhibition of SeMNPV superinfection but not of AcMNPV infection [26,38]. In this study, to understand the effect of latently infected baculovirus on insect cell cycle progression, flow cytometry was used to compare the cell cycle profiles of Se301 cells and latently infected P8-Se301-C1 cells, as well as the cell cycle characteristics of cells infected with homologous SeMNPV and heterologous AcMNPV. Further analysis of the sensitivity of P8-Se301-C1 cells in different cell cycle phases to SeMNPV superinfection showed that G1 phase infection and G2/M phase arrest could promote SeMNPV replication and, therefore, alleviate the inhibition of homologous virus superinfection. These findings will contribute to a better understanding of the molecular mechanisms by which latently infected viruses regulate cell cycle progression and provide new strategies for overcoming baculovirus superinfection exclusion and developing efficient baculovirus insecticides.

## 2. Materials and Methods

### 2.1. Cells and Virus Infection

The cells, including *S. exigua* Se301 cells, *S. frugiperda* Sf9 cells, and SeMNPV latently infected P8-Se301-C1 cells [26], were maintained at 27 °C in Grace’s medium (Gibco, Carlsbad, CA, USA) supplemented with 10% fetal bovine serum (FBS) (ExCell Bio, Shanghai, China) and a mixture of penicillin and streptomycin (100 μg/mL) (Solarbio, Beijing, China).

The SeMNPV US1 strain was propagated in *S. exigua* larvae following the method [39]. Briefly, fourth-instar larvae were fed with an artificial diet contaminated with SeMNPV OBs, and hemolymph was collected as the initial virus inoculum to infect Se301 cells. Four days after infection, the supernatant containing SeMNPV BV was used for subsequent virus infection.

The AcMNPV-recombinant virus vAc^PH-GFP^ was constructed by modifying the AcMNPV bacmid bMON14272 by insertion of the AcMNPV *polh* gene and the *enhanced green fluorescence protein* gene (*egfp*) into the *polh* locus [40]. The BAC/PAC DNA Kit (Omega Bio-Tek, Norcross, GA, USA) was used to extract vAc^PH-GFP^ bacmid DNA from *Escherichia coli* DH10B, and 2 μg of vAc^PH-GFP^ bacmid DNA was transfected into 2 × 10^6^ Sf9 cells. The cell supernatant containing vAc^PH-GFP^ BV at 96 h post-transfection (h p.t.) was collected as the initial virus inoculum to infect Sf9 cells, and the supernatant obtained 4 days after infection was used for subsequent virus infection.

Cells were infected with SeMNPV at an MOI of 1 or AcMNPV at an MOI of 10, respectively. After virus adsorption for 1 h at 27 °C, the cells were washed once with serum-free medium, and fresh medium was added (defined as 0 h post-infection, h p.i.). The infected cells were collected at the designated time points for subsequent experiments. Virus titers were determined by a tissue culture infectious dose 50% (TCID_50_) assay.

### 2.2. Cell Cycle Synchronization

The cell cycle inhibitors hydroxyurea and nocodazole (both from Sigma, Saint Louis, MO, USA) were used for cell synchronization. A total of 1 × 10^6^ cells (Se301 or P8-Se301-C1) were cultured in insect medium containing 80 μg/mL hydroxyurea for 20 h to synchronize the cells to the late G1 phase. The medium was removed, and the cells were washed twice with phosphate-buffered saline (PBS, pH 7.2). Fresh insect medium was added to release the cells for 6 h to synchronize G1 phase cells into the S-phase. After 18 h of release, P8-Se301-C1 cells were further cultured with a medium containing 7 μg/mL nocodazole for an additional 12 h to synchronize the cells in the G2/M phase.

### 2.3. Cell Cycle Analysis by Flow Cytometry

The cell cycle was determined by fluorescence-activated cell sorting (FACS) analysis after propidium iodide (PI) staining following the instructions of the PI Kit (BD, New York, NY, USA). PI is a kind of nucleic acid dye that labels DNA and emits red fluorescence, whose fluorescence intensity directly reflects the DNA content in cells. Because cells have various DNA contents in different cell cycle phases, the fluorescence intensity of DNA-bound PI detected by flow cytometry can distinguish each cell cycle phase into G0/G1, S, and G2/M phases. The cells (1 × 10^6^) were harvested, washed three times with PBS (pH 7.2), and then fixed with 70% pre-cooled ethanol at 4 °C for 24 h. After staining with PBS containing 50 µg/mL PI (BD, New York, NY, USA) and 20 µg/mL RNase for 30 min, the fluorescence intensity was analyzed by flow cytometry (FACSCanto, BD, New York, NY, USA). A minimum of 15,000 cell counts were performed for each sample. The flow cytometry histogram corresponding to each cell cycle was analyzed to calculate the percentage of cells in each phase using ModFit LT (version 5.0, Verity Software House). All experiments were carried out in triplicate.

### 2.4. Quantitative Real-Time PCR (qRT-PCR)

The transcriptional expression levels of cellular genes were analyzed by qRT-PCR. Se301 and P8-Se301-C1 cells (1 × 10^6^) were synchronized in the G1 phase by treatment with hydroxyurea and subsequently in the S phase by release for culture. The cells were harvested at different time points after releasing culture. Cellular RNA was extracted using an RNA-Quick Purification Kit (ESscience, Shanghai, China). RNA was then reverse transcribed into cDNA using the StarScript II RT Mix with gDNA Remover Kit (GenStar, Beijing, China). qRT-PCR was conducted using 2× RealStar Fast SYBR qPCR Mix (Low ROX) (GenStar, Beijing, China) and analyzed with the QuantStudio 3 Real-Time PCR Detection System (Applied Biosystems, Waltham, MA, USA). The PCR reaction program was as follows: 95 °C for 2 min, followed by 40 cycles of 95 °C for 15 s and 60 °C for 30 s.

The DNA replication-related genes and cell cycle regulatory genes, including *MCM4* (mini chromosome maintenance protein 4), *PCNA* (proliferating cell nuclear antigen), *BAF* (barrier-to-autointegration factor), *Cyclin B*, and *CDK1*, were amplified by PCR using Se301 cell DNA as a template, respectively. The PCR product was cloned into the pMD18-T vector (TaKaRa, Beijing, China) to generate plasmid pMD18-T-*MCM4/PCNA/BAF/Cyclin B/CDK1*. After transformation into *E. coli* DH5α, pMD18-T-*MCM4/PCNA/BAF/Cyclin B/CDK1* plasmid DNA was extracted using plasmid Mini Kit I (Omega Bio-Tek, Norcross, GA, USA). Then, 10× serial gradient dilution of the plasmid DNA was used as a template for qRT-PCR analysis. A standard curve was prepared from the respective cycle threshold (CT) value and the common log value (lg value) of the estimated DNA copies in 1 µL of the template. The absolute quantity standard curve was used to determine the gene transcription levels (Supplementary Figure S1A–E). The copy number of the DNA replication-related genes in the DNA genome at the indicated time points was calculated. All experiments were carried out in triplicate. The primer sequences used in target gene amplification are listed in Supplementary Table S1.

### 2.5. Viral Production Analysis in Infected Cells

The copy number of the essential gene *Se67* (a single-copy gene of SeMNPV) was quantified by qRT-PCR to determine the amount of virus or intracellular viral DNA content. The *Se67* gene was amplified by PCR using SeMNPV DNA as a template, and the PCR product was cloned into the pMD18-T vector (TaKaRa, Beijing, China) to generate plasmid pMD18-T-*Se67*. After transformation into *E. coli* DH5α, pMD18-T-*Se67* plasmid DNA was extracted using plasmid Mini Kit I (Omega Bio-Tek, Norcross, GA, USA). Then, a 10× serial gradient dilution of the plasmid DNA was used as a template for qRT-PCR analysis. The qRT-PCR reaction procedure was the same as described in 2.4 above. A standard curve was prepared from the Ct value and the common log value (lg value) of the *Se67* gene copy number (Supplementary Figure S1F).

P8-Se301-C1 cells (1 × 10^6^) were synchronized to the specific cell cycle phase by drug treatment, and the cells were then pre-cooled at 4 °C and then incubated with SeMNPV (MOI = 1) at 4 °C for 60 min. After the cells were washed twice with cold PBS to remove the unbound virus, the total cellular DNA was extracted using the MiniBEST Viral RNA/DNA Extraction Kit Ver. 5.0 kit (TaKaRa, Beijing, China) and used as the template for qRT-PCR analysis. According to the Ct value detected by qRT-PCR analysis and the standard curve of pMD18-T-*Se67* plasmid DNA, the copy number of the viral DNA genome was calculated to determine the amount of virus adsorbed on the cell surface. All experiments were carried out in triplicate.

P8-Se301-C1 cells (1 × 10^6^) were synchronized to the specific cell cycle phase by drug treatment and were then infected with SeMNPV (MOI = 1) for 48 h at 27 °C. The infected cells were harvested, and the total cellular DNA was extracted as the template for qRT-PCR analysis. According to the Ct value detected by qRT-PCR analysis and the standard curve of pMD18-T-*Se67* plasmid DNA, the copy number of the viral DNA genome was calculated to analyze viral genomic DNA replication. All experiments were carried out in triplicate.

P8-Se301-C1 cells (1 × 10^6^) were synchronized to the specific cell cycle phase by drug treatment and then infected with SeMNPV (MOI = 1) at 27 °C. At the indicated time points, the morphological and cytopathic effects of the cells were observed by phase contrast microscopy. The culture supernatant containing BVs was harvested, and the BV genomic DNA of 100 μL of supernatant was extracted using the MiniBEST Viral RNA/DNA Extraction Kit Ver.5.0 kit (TaKaRa, Beijing, China). According to the Ct value detected by qRT-PCR analysis and the standard curve of pMD18-T-*Se67* plasmid DNA, the copy number of the viral DNA genome was calculated to analyze the progeny BV yield in the supernatant. All experiments were carried out in triplicate.

### 2.6. Statistical Analysis

Data are presented as the mean ± standard deviation of three independent experiments. A one-way analysis of variance (ANOVA) was used for statistical analysis. For each experiment, a Student’s *t*-test was used for statistical comparison, and a value of *p* < 0.05 was considered significant.

## 3. Results

### 3.1. Cell Cycle Distribution and Differences between Se301 and P8-Se301-C1 Cells

**Figure 1:**
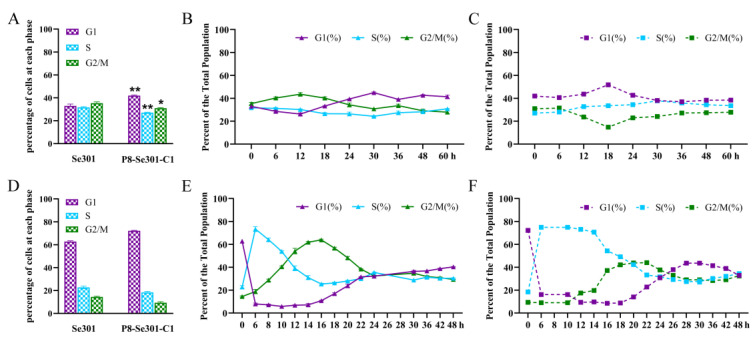
Cell cycle distribution and differences between Se301 and P8-Se301-C1 cells. Se301 and P8-Se 301-C1 cells (1 × 10^6^) were seeded in 60 mm diameter dishes, and then cells were harvested at indicated time points. After being stained with PI, the cell cycle distribution in the G1, S, and G2/M phases was determined by flow cytometry. (**A**) Cell cycle distribution of Se301 and P8-Se301-C1 cells at 0 h after subculture. Cell cycle distribution of Se301 cells (**B**) and P8-Se301-C1 cells (**C**) at indicated time points after subculture. The cells (1 × 10^6^) were treated with 80 μg/mL hydroxyurea for 20 h to synchronize in the G1 phase, and the cell cycle distribution of Se301 and P8-Se301-C1 cells was determined (**D**). Then, the G1 phase synchronized cells were released into culture with fresh medium, and the cell cycle progress of Se301 cells (**E**) and P8-Se301-C1 cells (**F**) at the indicated time points after release culture were determined. Data are presented as mean ± standard deviation from triplicate biological experiments. * *p* <0.05, ** *p* < 0.01.

The cell cycle distribution of Se301 cells and P8-Se301-C1 cells was determined using flow cytometry. The results showed that the two cell types showed different cell cycle distributions during culture (Supplementary Figure S2). After subculture (0 h), the proportion of P8-Se301-C1 cells in the G1 phase (41.97 ± 0.48%) was significantly higher than that of Se301 cells (32.95 ± 1.67%) (*p* < 0.01). Moreover, the proportions of S phase cells (27.04 ± 0.47%) and G2/M phase cells (30.9 ± 0.38%) in P8-Se301-C1 were significantly lower than those in Se301 cells (31.69 ± 0.60% and 35.36 ± 1.07%, respectively) (*p* < 0.05) (Figure 1A). At 30 h after subculture, the proportion of Se301 cells in the S phase decreased to 24.27 ± 0.66%, while the proportion of P8-Se301-C1 cells in the S phase increased to 37.85 ± 0.71% (Figure 1B,C), implying a different progression between Se301 cells and P8-Se301-C1 cells in the S phase.

Se301 and P8-Se301-C1 cells were arrested at the late G1 phase by hydroxyurea treatment to further analyze cell cycle characteristics (Figure 1D). Upon release culture, the number of synchronized cells in the G1 phase rapidly declined and accumulated in the S phase within 6 h (Se301 cells) or 12 h (P8-Se301-C1 cells). As the culture progressed, cells in the S phase gradually decreased, while cells in the G2/M phase began to accumulate and reached a peak at 14–16 h (Se301 cells) or 20–22 h (P8-Se301-C1 cells) after release, respectively (Figure 1E,F, Supplementary Figure S3). The results indicate a 6 h difference between Se301 and P8-Se301-C1 cells in the progression time from late G1 to G2/M phase (S phase).

### 3.2. Transcription Analysis of DNA Replication-Related Genes in Se301 and P8-Se301-C1 Cells

**Figure 2:**
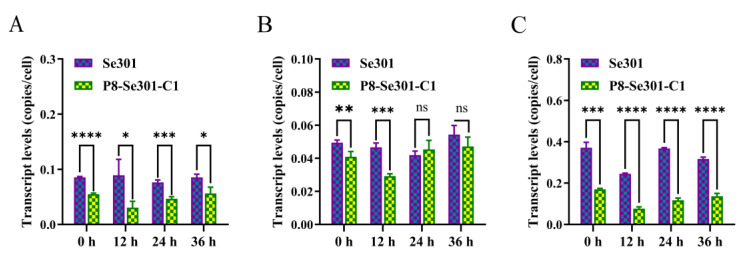
Transcription analysis of DNA replication-related genes in Se301 and P8-Se301-C1 cells. Cells (1 × 10^6^ cells) were seeded in 25 mm diameter dishes and synchronized in the G1 phase by treatment with hydroxyurea and subsequently in the S phase by releasing for culture. At different time points after releasing culture, the transcription levels of *MCM4* (**A**), PCNA (**B**), and *BAF* (**C**) in Se301 and P8-Se301-C1 cells were detected by qRT-PCR. Data are presented as mean ± standard deviation in biological triplicate experiments. ns: no significant, * *p* < 0.05, ** *p* < 0.01, *** *p* < 0.001, **** *p* < 0.0001.

To further clarify the difference in S phase progression between Se301 cells and P8-Se301-C1 cells, the cells were synchronized in the G1 phase and subsequently in the S phase by releasing for culture, and the transcription levels of key genes involved in DNA replication were examined. The results of qRT-PCR analysis showed that the transcription levels of *MCM4*, *PCNA* (at 0 h and 12 h after releasing culture), and *BAF* in P8-Se301-C1 cells were markedly lower than those in Se301 cells (*p* < 0.05) in S phase (Figure 2). This suggests that the slower progression of the S phase in P8-Se301-C1 cells may be attributed to the down-regulation of DNA replication-related genes.

### 3.3. Effects of a Homologous Virus SeMNPV and Heterologous Virus AcMNPV Infection Affects the Cell Cycle of Se301 and P8-Se301-C1 Cells

**Figure 3:**
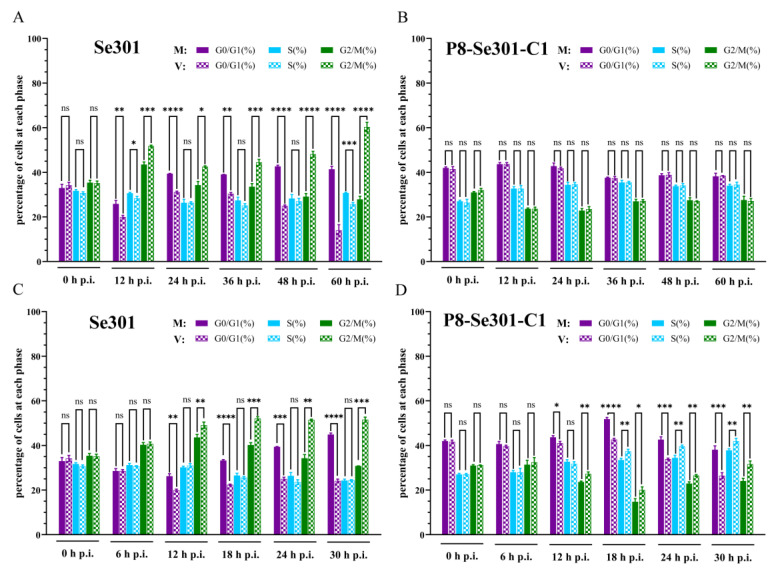
Effects of a homologous virus SeMNPV and heterologous virus AcMNPV infection affects the cell cycle of Se301 and P8-Se301-C1 cells. Cell cycle distribution of Se301 (**A**) and P8-Se301-C1 (**B**) after infection with SeMNPV. Cell cycle distribution of Se301 cells (**C**) and P8-Se301-C1 cells (**D**) after infection with vAc^PH-GFP^. Cells were infected with SeMNPV at an MOI of 1 or vAc^PH-GFP^ at an MOI of 10. Mock infections were performed by replacing the viral supernatant with the medium. At the indicated time points after infection, the cells were harvested and stained with PI, and the cell cycle distribution was analyzed by flow cytometry. V: virus-infected cells; M: mock-infected cells. Data are presented as mean ± standard deviation in biological triplicate experiments. ns: no significant, * *p* < 0.05, ** *p* < 0.01, *** *p* < 0.001, **** *p* < 0.0001.

To investigate the effect of baculovirus infection on the cell cycle, Se301 cells and P8-Se301-C1 cells were infected with homologous virus SeMNPV and heterologous baculovirus vAc^PH-GFP^ (a recombinant AcMNPV), respectively (Supplementary Figure S4). Flow cytometry analysis revealed that, compared with the mock-infected cells, both SeMNPV and vAc^PH-GFP^-infected Se301 cells resulted in a significant increase in the proportion of cells in the G2/M phase since 12 h p.i. (*p* < 0.05) (Figure 3A,C). However, SeMNPV infection did not induce any changes in the cell cycle distribution of P8-Se301-C1 cells (Figure 3B). In addition, vAc^PH-GFP^ infection induced G2/M and S phase arrest in P8-Se301-C1 cells (Figure 3D). These results demonstrate that both SeMNPV and vAc^PH-GFP^ can induce G2/M phase arrest in Se301 cells, while heterologous vAc^PH-GFP^, but not homologous SeMNPV, can induce cell cycle arrest in P8-Se301-C1 cells.

### 3.4. P8-Se301-C1 Cells in G1 Phase Were More Susceptible to SeMNPV Superinfection

**Figure 4:**
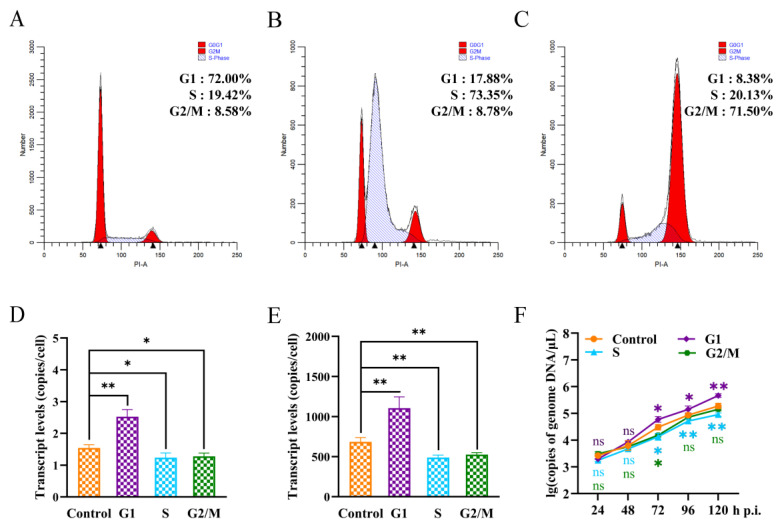
Sensitivity of P8-Se301-C1 cells at different cell cycle phases to SeMNPV superinfection. The flow cytometry histograms show that P8-Se301-C1 cells were synchronized in (**A**) G1 phase (cultured in medium containing 80 μg/mL hydroxyurea for 20 h); (**B**) S phase (cultured in medium containing 80 μg/mL hydroxyurea for 20 h and released for 6 h); (**C**) and G2/M phase (cultured in medium containing 80 μg/mL hydroxyurea for 20 h and released for 18 h, and then cultured in medium containing 7 μg/mL nocodazole for 12 h), respectively. After the cells were infected with SeMNPV (MOI = 1) at 0 °C or 27 °C, qRT-PCR was performed to analyze the amount of virus adsorbed on the cell surface at 0 h p.i. (**D**), intracellular viral DNA replication at 48 h p.i. (**E**), and the BV production of the culture supernatant at indicated time points (**F**). The unsynchronized P8-Se301-C1 cells were used as a control. Data are presented as mean ± standard deviation in biological triplicate experiments. ns indicates non-significant; * *p* < 0.05, ** *p* < 0.01.

In order to reveal the response of different specific cell cycle phases to SeMNPV superinfection, P8-Se301-C1 cells were synchronized to G1 (72.00%), S (73.35%), and G2/M (71.50%) phases (Figure 4A–C), respectively, and then were infected with SeMNPV. The adsorption of viral particles on the cell surface, the replication of intracellular viral DNA, and the viral production of progeny were quantitatively analyzed by qRT-PCR. Compared with unsynchronized cells, the amount of virus adsorption and intracellular viral DNA replication in G1 phase cells were significantly increased (*p* < 0.05), while those in S and G2/M phase cells were significantly decreased (*p* < 0.05) at 48 h p.i. (Figure 4D,E). In addition, the detection of BV production in the supernatant of infected cells revealed that the BV production in synchronized cells in G1, S, and G2/M phases was comparable to that in unsynchronized cells at 24 h p.i. (*p* > 0.05). However, from 72 h p.i. to 120 h p.i., the BV production in the G1 phase cells was significantly higher than that in unsynchronized cells (*p* < 0.05), while the BV production in the S and G2/M phase cells decreased from 72 h p.i. (Figure 4F). These results suggested that the sensitivity of P8-Se301-C1 cells in G1, S, and G2/M phases to SeMNPV infection was different. The G1 phase was more advantageous to SeMNPV infection and could partially alleviate the exclusion effect of SeMNPV superinfection in P8-Se301-C1 cells.

### 3.5. SeMNPV Superinfection of G1 Phase P8-Se301-C1 Cells Induced G2/M Phase Arrest and Downregulated Cyclin B and CDK1 Expression

**Figure 5:**
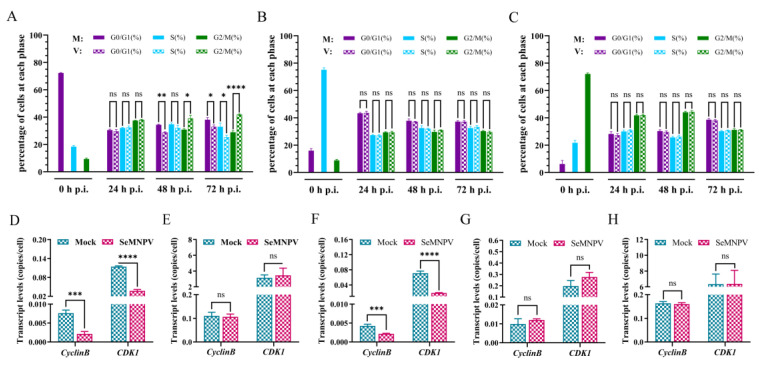
SeMNPV superinfection of G1-phase P8-Se301-C1 cells induced G2/M-phase arrest and downregulated *Cyclin B* and *CDK1* expression. After P8-Se301-C1 cells were infected with SeMNPV, flow cytometry analysis was performed to determine the cell cycle distributions at the indicated time points. Histograms show the percentage of cells in each phase at different times of SeMNPV infection of P8-Se301-C1 cells synchronized to the G1 phase (**A**), synchronized to the S phase (**B**), and synchronized to the G2/M phase (**C**). qRT-PCR analysis was used to determine the transcriptional levels of *CyclinB* and *CDK1* in SeMNPV-infected unsynchronized Se301 cells (**D**), unsynchronized P8-Se301-C1 cells (**E**), G1 phase (**F**), S phase (**G**), and G2/M phase P8-Se301-C1 cells (**H**). Data are presented as mean ± standard deviation in biological triplicate experiments. ns: no significant, * *p* < 0.05, ** *p* < 0.01, *** *p* < 0.001, **** *p* < 0.0001.

The cell cycle arrest of SeMNPV-infected P8-Se301-C1 cells at different cell cycle phases was analyzed (Supplementary Figure S5). Flow cytometry analysis showed that SeMNPV infection of P8-Se301-C1 cells in the G1 phase did not cause significant changes in the cell cycle within 24 h p.i. However, the proportion of G2/M phase increased in the infected cells was higher than that in the mock-infected cells at 48 h p.i. (*p* < 0.05) and continued to increase until 72 h p.i. (Figure 5A). Nevertheless, SeMNPV infection of P8-Se301-C1 cells in either the S phase or the G2/M phase did not result in considerable differences in cell cycle distribution (Figure 5B,C). These results suggest that SeMNPV could induce the accumulation of P8-Se301-C1 cells in the G2/M phase to a certain extent by infecting cells in the G1 phase rather than the S or G2/M phase.

Cyclin B and CDK1 are key factors regulating G2 phase progression and M phase entry in cells. The results of qRT-PCR analysis showed that compared to the mock-infected cells, the transcription levels of *Cyclin B* and *CDK1* were significantly downregulated in Se301 cells and G1 phase P8-Se301-C1 cells 48 h after SeMNPV infection (*p* < 0.05) (Figure 5D,F). However, the transcription levels of *Cyclin B* and *CDK1* did not change significantly after infection of unsynchronized, S-phase, or G2/M phase P8-Se301-C1 cells (Figure 5E,G,H). The results showed that infection of G1-phase P8-Se301-C1 cells with SeMNPV inhibited *Cyclin B* and *CDK1* expression, as did infection of Se301 cells.

### 3.6. G2/M Phase Arrest Was Required for SeMNPV Replication in P8-Se301-C1 Cells

**Figure 6:**
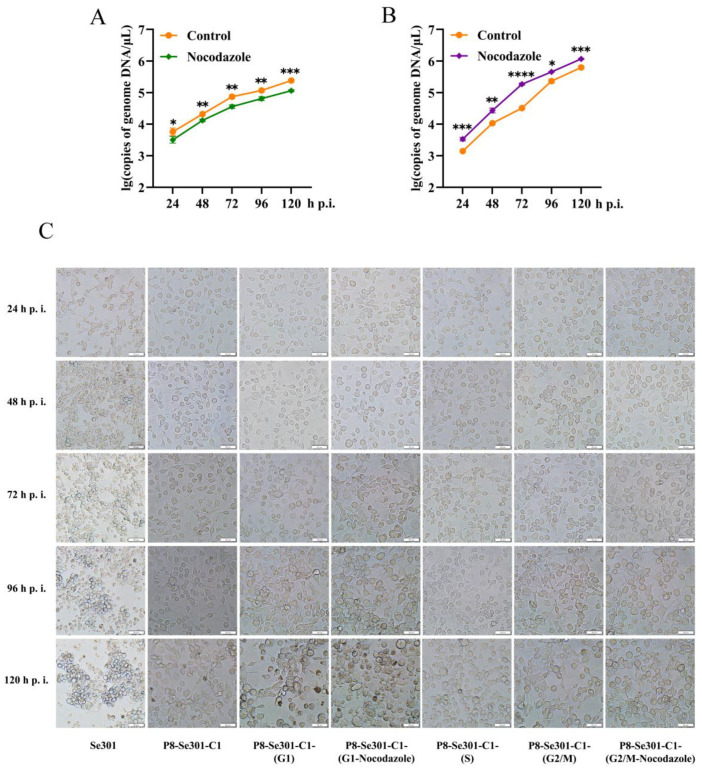
G2/M phase arrest is required for SeMNPV replication in P8-Se301-C1 cells. P8-Se301-C1 cells synchronized to G2/M (**A**) and G1 (**B**) phases were infected with SeMNPV (MOI = 1) for 1 h and then cultured in a medium containing 7 μg/mL nocodazole to arrest cells in the G2/M phase. qRT-PCR was performed to measure the BV production in the culture supernatant at the indicated time points. The infected cells cultured in a medium without nocoodazole were used as the control. (**C**) Light microscopy of SeMNPV infected P8-Se301-C1 cells at specific cell cycle phases. P8-Se301-C1 cells were synchronized at the G1 phase, S phase, and G2/M phase and then infected with SeMNPV (MOI = 1). In addition, SeMNPV-infected G1 and G2/M cells were treated with nocodazole to further arrest the infected cells in the G2/M cells. SeMNPV-infected, unsynchronized Se301 and P8-Se301-C1 cells were used as controls. Bar = 50 μm. Data are presented as the mean ± standard deviation in biological triplicate experiments. * *p* < 0.05, ** *p* < 0.01, *** *p* < 0.001, **** *p* < 0.0001.

To understand whether G2/M phase arrest is essential for virus multiplication in P8-Se301-C1 cells, progeny virus production was determined by infecting P8-Se301-C1 cells in G2/M phase with SeMNPV and subsequently arresting cells in G2/M phase with nocodazole treatment. SeMNPV-infected cells without nocodazole treatment were used as controls. The results of qRT-PCR analysis indicated that BV production was not increased but decreased compared with control cells (*p* < 0.05) (Figure 6A). In contrast, when P8-Se301-C1 cells were infected in the G1 phase and then arrested in the G2/M phase by nocodazole treatment, the production of BV significantly increased (*p* < 0.05) (Figure 6B). It was suggested that G1-phase infection and G2/M arrest are both necessary for SeMNPV multiplication in P8-Se301-C1 cells.

Light microscopy showed that no significant morphological differences were observed in SeMNPV-infected P8-Se301-C1 cells at G1, S, and G2/M phases at 24 h p.i. At 96 h p.i., after infection of unsynchronized P8-Se301-C1 cells with SeMNPV, the cells showed mild cytopathic effects, but cells containing OBs were not observed. In contrast, cells infected in the G1 phase showed more apparent cytopathic effects (with or without nocodazole treatment), most of which became round, enlarged, aggregated, and formed multicellular. Moreover, polyhedra appeared in a few cells, and the number of OBs-containing cells in infected cells treated with nocodazole (about 10%) was higher than that in untreated infected cells (less than 5%). At 120 h p.i., less than 10% of the infected, unsynchronized P8-Se301-C1 cells contained OBs. In contrast, of the G1 phase-infected cells, the OBs-containing cells in the nocodazole-treated and nocodazole-untreated cells are about 24.1% and 14.1%, respectively. In addition, until 120 h p.i., no polyhedra were observed in SeMNPV-infected cells in S-phase and G2/M-phase cells (with or without nocodazole treatment), except for cytopathic effects such as cell rounding and aggregation (Figure 6C).

## 4. Discussion

Many viral infections can modulate the cell cycle, leading to changes in cell cycle progression that promote viral replication. Hijacking and altering the cell cycle are essential for the virus to establish and maintain a latent infection [31,36]. In this study, we reveal that baculovirus latent infection interfered with the cell cycle progression. SeMNPV superinfection failed to induce cell cycle arrest in P8-Se301-C1 cells. Furthermore, specific G1-phase infection and G2/M-phase arrest favoring SeMNPV replication are effective ways to alleviate SeMNPV superinfection exclusion in P8-Se301-C1 cells. This study contributes to a better understanding of the interplay between baculovirus latent infection and host cell cycle regulation and provides new insights into the mechanism of overcoming antiviral effects.

The cell cycle distribution of *S. exigua* cells was comparable in proportions to G1, S, and G2/M phases (Figure 1D), similar to that of *S. frugiperda* Sf9 cells [41]. In contrast, the cell cycle distributions of *Helicoverpa armigera* Hz-AM1 cells and *B. mori* BmN-SWU1 cells showed differences in cell cycle distribution, with G1 phase cells predominating in the former and G2/M phase cells in the latter [16,42]. Cell cycle distribution and progression can vary owing to biological characteristic differences in cell types or culture methods [43,44,45].

The cell cycle progression of SeMNPV-latently infected P8-Se301-C1 cells was perturbed. This result of cell cycle prolongation in P8-Se301-C1 cells (Figure 1E,F) is consistent with our previous study, showing that P8-Se301-C1 cells have a slower growth rate [26]. The prolonged S phase of P8-Se301-C1 cells may be related to impaired DNA synthesis. The transcription levels of host DNA replication-related genes, *MCM4*, *PCNA*, and *BAF*, in P8-Se301-C1 cells were significantly lower than those in Se301 cells (Figure 2). MCM forms a heterohexamer (MCM2-7) to initiate DNA replication in the G1/S phase, while BAF binding to PCNA promotes DNA replication in the S phase [46,47]. Previous studies have shown that transient inhibition of MCM and PCNA transcription does not affect cell cycle progression from late G1 into the S phase but delays S phase exit [48]. RNAi knockdown of BAF directly mediates S phase arrest in cells [47]. In addition, both cellular and viral DNA replication-related genes are involved in viral replication; for example, AcMNPV utilizes both cellular and viral PCNAs in its DNA replication, whereas *pcna*-defective AcMNPV mutants substitute cellular PCNA for viral PCNA [49]. The regulation of baculovirus latency-associated genes or transcripts on host cell DNA replication and the cell cycle needs to be further elucidated.

Compared with the S and G2/M phases, G1 phase P8-Se301-C1 cells were more susceptible to SeMNPV infection, and the production of progeny BV and OB increased after SeMNPV infection (Figure 4F and Figure 6C). The infection efficiency of the virus varies among cells due to the different physiological states of cells at different phases of the cell cycle, including cell activity [20] and the expression of virus receptors on the cell membrane surface [50]. Previous studies have shown that AcMNPV infection of Sf9 cells in the G1 phase was more efficient, enhanced the expression of recombinant proteins [19], and increased the production of BV and OB [20]. The amount of virus adsorption and intracellular viral DNA replication were significantly increased in G1 phase cells compared with unsynchronized cells and cells in S and G2/M phases (Figure 4D,E). The G1 phase is the preparatory phase for cellular DNA replication, during which cellular metabolism is active [51] and membrane transport is enhanced [52,53], which may facilitate viral nucleocapsid transport across the cell membrane to the nucleus for replication [54]. Therefore, SeMNPV infection in the G1 phase of P8-Se301-C1 cells may promote progeny BV production by increasing the amount of virus adsorbed on the cell surface and viral DNA replication.

Infection of Se301 cells with SeMNPV and vAc^PH-GFP^ resulted in G2/M phase arrest (Figure 3A,C), consistent with AcMNPV, HaSNPV, and BmNPV infecting their permissive cells [16,41,42], suggesting that inducing G2/M phase cell cycle arrest may be a common strategy for baculovirus infection. G2/M phase cell cycle arrest can maintain cells in a “pseudo-S phase,” which is more conducive to virus replication, thus preventing viral replication from competing with cellular DNA replication for nucleotide libraries and reducing the virus’s reliance on the S phase environment [55,56]. Moreover, the transcriptional suppression of mitotic genes in G2/M phase cells can inhibit the expression of antiviral genes, facilitating viral replication and proliferation [12]. Furthermore, G2/M phase arrest may also be advantageous for viral transport due to the availability of microtubules or mitotic spindles [10,11].

However, SeMNPV infection did not cause G2/M phase arrest in latently infected P8-Se301-C1 cells (Figure 3B). Therefore, while P8-Se301-C1 cells were arrested in the G2/M phase by infecting in the specific G1 phase (Figure 5A), it may create a favorable environment for SeMNPV multiplication and thus alleviate the SeMNPV superinfection exclusion. SeMNPV infection in G1-phase P8-Se301-C1 cells could inhibit the transcription levels of *Cyclin B* and *CDK1* (Figure 5F). Cyclin B and CDK1 bind to form a maturation-promoting factor (MPF), which drives cells from the G2 to M phase by phosphorylating substrate proteins [57]. Virus infection can reduce *Cyclin B* and *CDK1* expression [16] or inhibit Cyclin B1-CDK1 complex formation and nuclear import [58]. Therefore, these results suggest that SeMNPV infection failure to induce G2/M phase arrest in latently infected cells may be the key event leading to homologous superinfection exclusion.

Nonetheless, G2/M phase arrest alone was not sufficient to relieve the inhibition of SeMNPV superinfection on P8-Se301-C1 cells. Previous studies have indicated that G2/M phase arrest induced by nocodazole treatment in BmN-SWU1 cells promotes BmNPV virus proliferation [16]. However, only infection in the G1 phase and arrest in the G2/M phase enhanced BV and OB production (Figure 6B,C), suggesting that G2/M phase arrest and G1 phase initial infection are beneficial for SeMNPV replication in P8-Se301-C1 cells. Viral infection is associated with viral receptors on the cell membrane surface [59]. The interaction between the virus adsorption protein and the host cell surface adsorption receptor, which serves as the rate-limiting step of virus infection, plays an important role in the process of virus infection [34]. We recently clarified that the superinfection exclusion of SeMNPV in P8-Se301-C1 cells has occurred in the adsorption stage. So, it is reasonably suggested that the presence of viral genomes and transcripts in P8-Se301-C1 cells [38] may lead to a reduction in the amount of virus absorbed on the cell surface, which may be one of the reasons affecting its inhibition of SeMNPV superinfection. Moreover, the amount of virus adsorption increased after SeMNPV infection of G1 phase cells (Figure 4D), which may be critical for successful superinfection of SeMNPV in P8-Se301-C1 cells. Future studies on the regulation and interaction of host and viral genes and cell surface receptors related to latent infection will help to further reveal the homologous superinfection exclusion and the mechanism of baculovirus latent infection.

**Figure 7:**
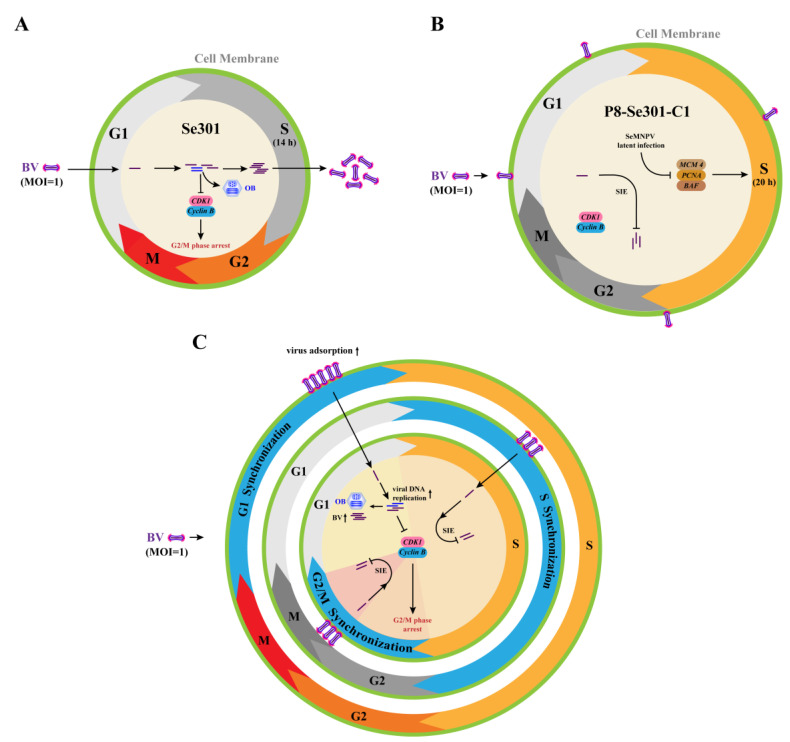
Schematic of SeMNPV superinfection exclusion alleviation by cell cycle progression regulation in latently infected P8-Se301-C1 cells. (**A**) Cell cycle progression of Se301 cells infected with SeMNPV. SeMNPV infection of Se301 cells downregulated the expression of *Cyclin B* and *CDK1*, induced G2/M phase arrest, and produced progeny BVs and ODVs. (**B**) Regulation of cell cycle progression in P8-Se301-C1 by SeMNPV superinfection. The expression of DNA replication-related genes *MCM 4*, *PCNA*, and *BAF* was downregulated, and the S phase was prolonged in P8-Se301-C1 cells. SeMNPV superinfection did not change *Cyclin B* and *CDK1* expression or induce G2/M phase arrest, and viral progeny production was inhibited. (**C**) SeMNPV infection of G1-phase P8-Se301-C1 cells regulated cell cycle progression and partially alleviated SeMNPV superinfection exclusion. SeMNPV superinfection of G1-phase P8-Se301-C1 cells promoted viral adsorption on the cell surface and intracellular viral DNA replication, downregulated *Cyclin B* and *CDK1* expression, induced G2/M phase arrest, and increased the production of BVs and OBs. Synchronization of SeMNPV-infected G1 phase P8-Se301-C1 cells in the G2/M phase further increased progeny virus production. However, SeMNPV infection of S-phase or G2/M-phase P8-Se301-C1 cells did not downregulate *Cyclin B* and *CDK1* expression, and the production of progeny BVs and OBs was deduced.

Taken together, this study revealed that the cell cycle distribution and S phase duration in SeMNPV-latently infected P8-Se301-C1 cells were different from those in Se301 cells. Infection of P8-Se301-C1 cells with SeMNPV did not induce G2/M phase arrest as did infection of Se301 cells. When P8-Se301-C1 cells were synchronized in the G1 phase, SeMNPV superinfection could downregulate the expression of *Cyclin B* and *CDK1* and induce G2/M phase arrest. In addition, G1 phase infection promoted the amount of viral adsorption on the cell surface and intracellular viral DNA replication of superinfected SeMNPV and increased the production of BVs and OBs. Moreover, synchronization of SeMNPV-infected G1-phase P8-Se301-C1 cells in the G2/M phase further increased the progeny virus production (Figure 7). In conclusion, we have proposed an effective strategy to alleviate homologous baculovirus superinfection exclusion in latently infected cells by specific G1 phase infection and G2/M phase arrest.

## Data Availability

Data are contained within the article and Supplementary Materials.

## Supplementary Materials

The following supporting information can be downloaded at https://www.mdpi.com/article/10.3390/v16050736/s1, Table S1. Primer used in this study. Figure S1. Standard curve of genes copy number. Figure S2. Cell cycle distribution of Se301 and P8-Se301-C1 cells. Figure S3. Cell cycle progression of Se301 and P8-Se301-C1 cells. Figure S4. Cell cycle analysis of Se301 and P8-Se301-C1 cells infected by the homologous virus SeMNPV and the heterologous virus AcMNPV. Figure S5. Cell cycle analysis of SeMNPV superinfected of synchronized P8-Se301-C1 cells.
