# Supplementary material for: Partial Alleviation of Homologous Superinfection Exclusion of SeMNPV Latently Infected Cells by G1 Phase Infection and G2/M Phase Arrest
Source: Viruses. 2024 May 6;16(5):736. doi: 10.3390/v16050736 (PMC11126141; doi:10.3390/v16050736)
Supplement: Supplementary file 1 [file viruses-16-00736-s001.zip › Supplementary Material.docx]

Supplementary Material

**Supplementary** Table S1. Primer sequences were used in this study.

| Gene | Forward primer (5' → 3') | Reverse primer (5' → 3') |
| --- | --- | --- |
| *MCM4* | CGAAGGCGGGTATCATCTGT | ATACCTCGTCTTGTGGGTCC |
| *PCNA* | AGGAGGAGGAGGCTGTTGTT | TGCGGTACTCAACCACAAGT |
| *BAF* | CGTCGGTGAGGTATTGGGAA | TGTCCTTCAGCCATTCTTGGA |
| *Se67* | CCGAGACCCATTTGAACGGT | GCAGTCGGTCGTTTGTCTGA |
| *Cyclin B* | AAGGTTTGACTGTGCGTGGA | GCCCGATTTAGTGTCGCCTT |
| *CDK1* | GCCAGACTACAAGCCCACAT | TCACATCGCGGAAGTATCGG |


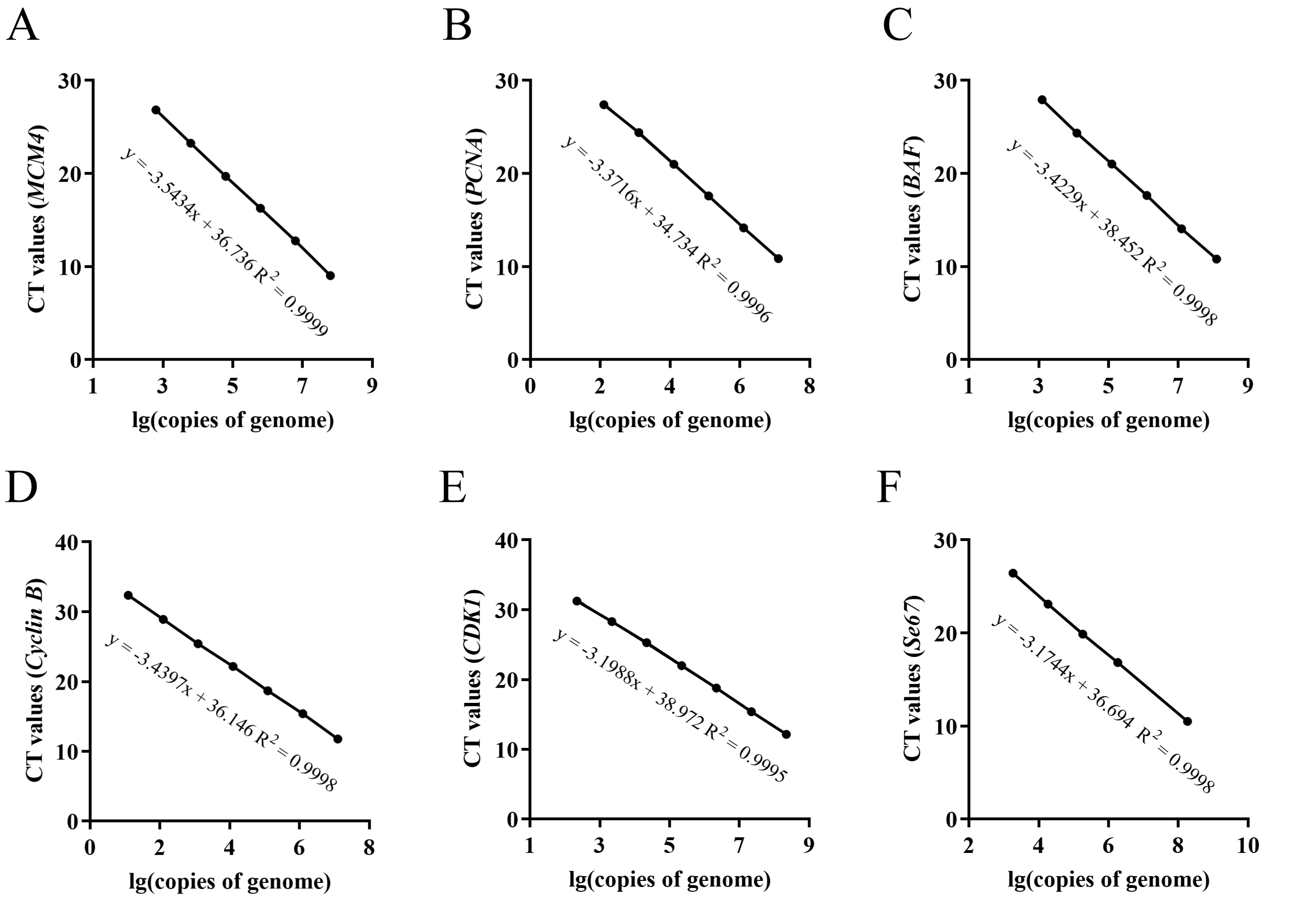


Figure S1. Standard curve of genes copy number. The target gene fragments were cloned into the pMD18-T vector and then transformed into *E. coli* DH5α, and the plasmid DNA was extracted. Plasmid DNA with a serial gradient dilution of 10 was used as a template for qRT-PCR analysis. Standard curves were prepared based on Ct values and common logarithmic values (lg values) of genes *MNM4* (A), *PCNA* (B), *BAF* (C), *Cyclin B* (D), *CDK1* (E) and *Se67* (F) copies.


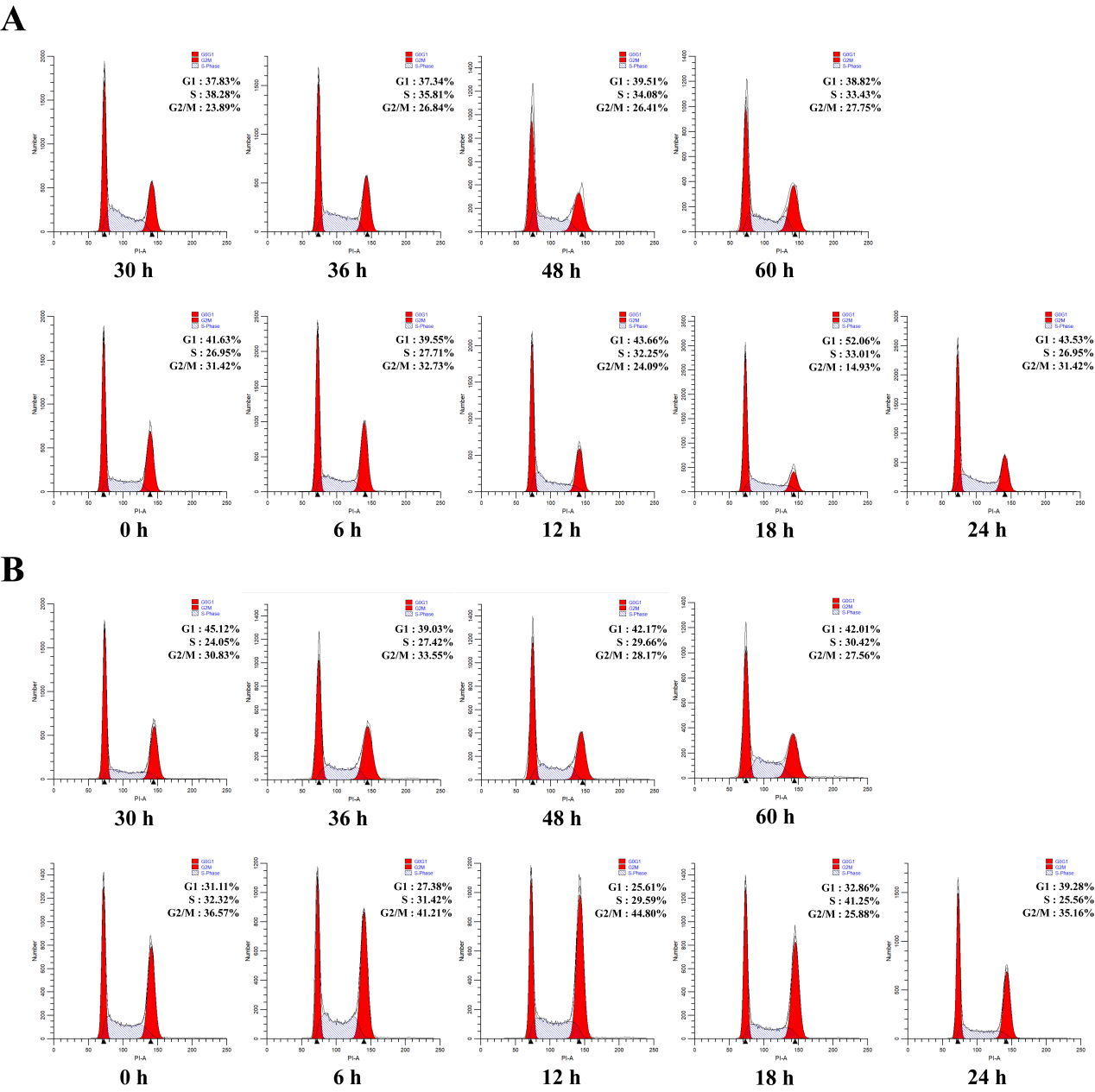


Figure S2. Cell cycle distribution of Se301 and P8-Se301-C1 cells. Cell cycle distribution of Se301 cells (A) and P8-Se301-C1 cells (B) at indicated time points after subculture. The cells (1×10^6^) were seeded in 60-mm-diameter dishes and then harvested at the indicated time points. After being stained with PI, the distribution of the cells in the G1, S, and G2/M phases was determined by flow cytometry.


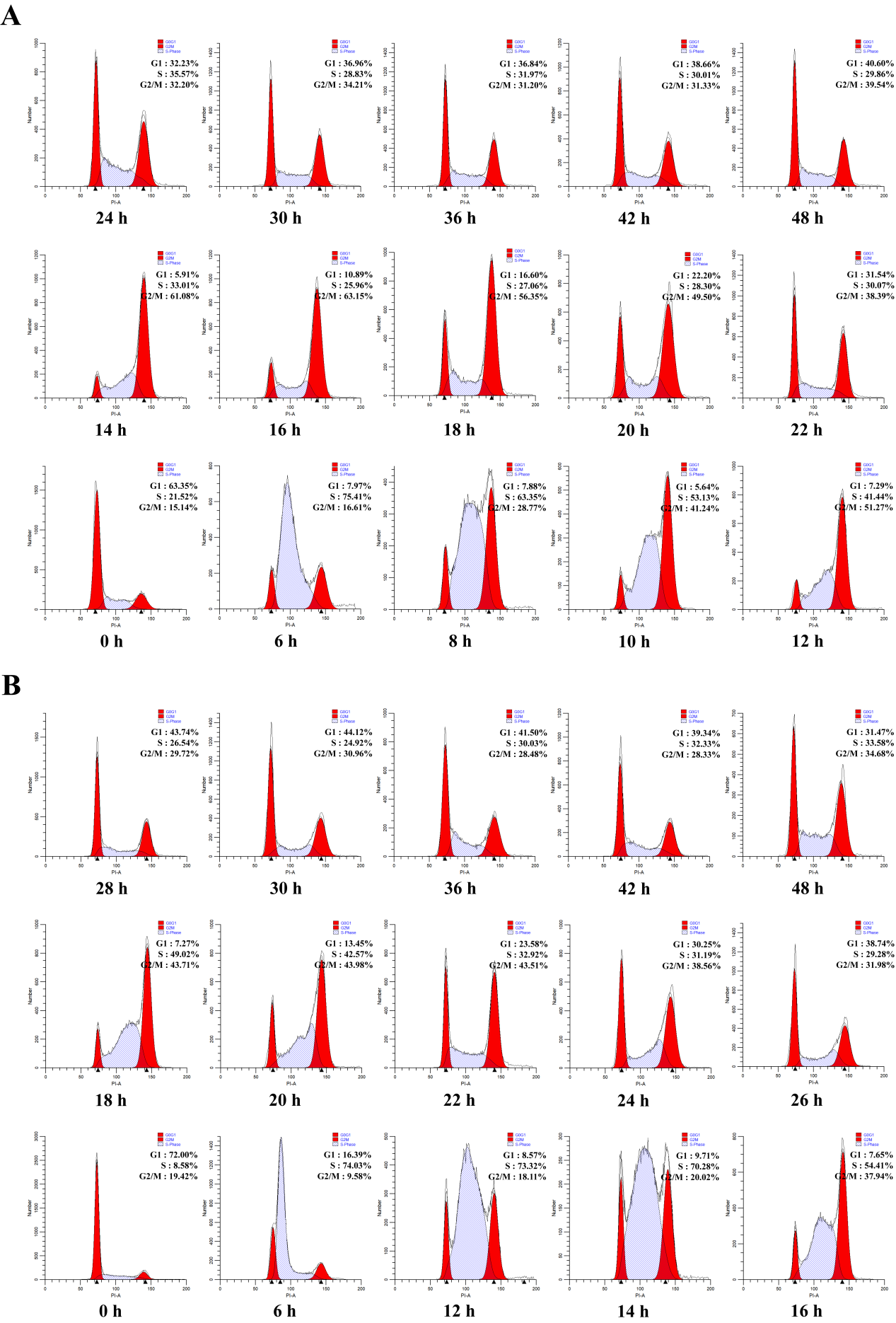


Figure S3. Cell cycle progression of Se301 and P8-Se301-C1 cells. The cells (1×10^6^) were treated with 80 μg/mL hydroxyurea for 20 h to synchronize in the G1 phase, then fresh medium cultured cells to determine the cell cycle distribution of Se301 cells (A) and P8-Se301-C1 cells (B) at the indicated time points after release culture.


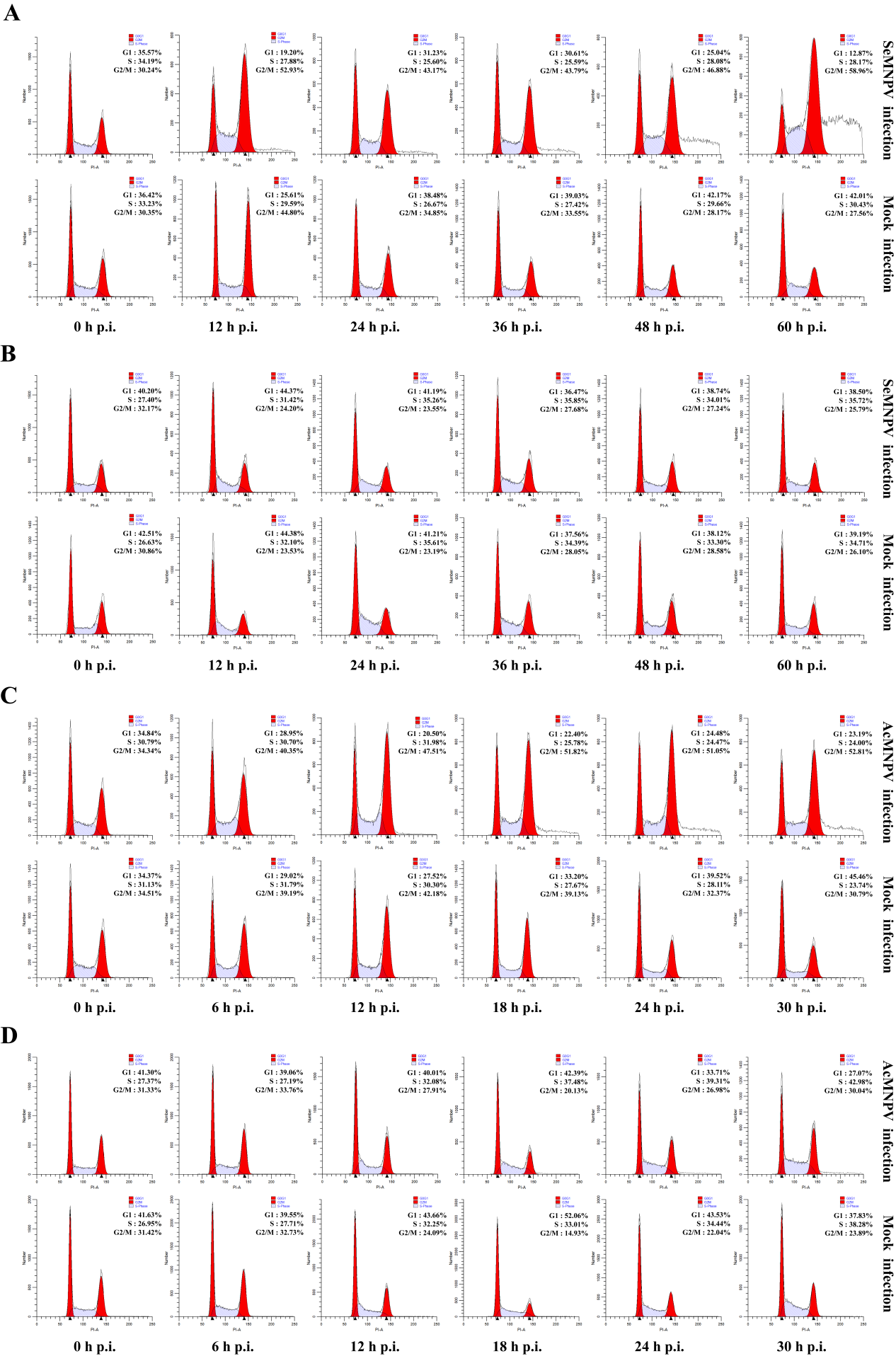


Figure S4. Cell cycle analysis of Se301 and P8-Se301-C1 cells infected by the homologous virus SeMNPV and the heterologous virus AcMNPV. SeMNPV infected Se301 cells (**A**), SeMNPV infected P8-Se301-C1 cells (**B**), vAc^PH-GFP^ infected Se301 cells (**C**), vAcPH-GFP infected P8-Se301-C1 cells (**D**). Cells were infected with SeMNPV at an MOI of 1 or vAc^PH-GFP^ at an MOI of 10. Mock infections were performed by replacing the viral supernatant with the medium.


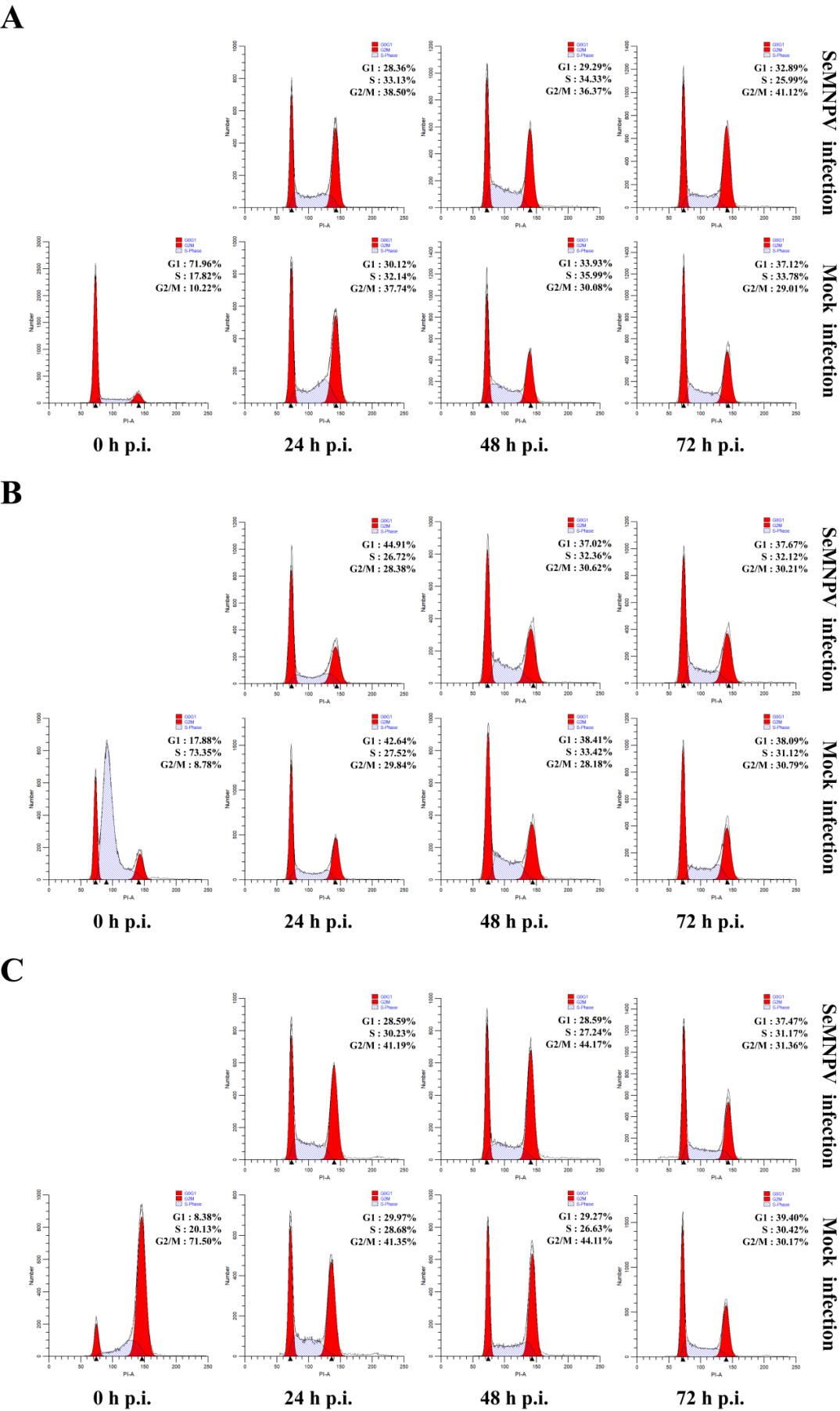


Figure S5. Cell cycle analysis of SeMNPV superinfected of synchronized P8-Se301-C1 cells. Cell cycle distribution of P8-Se301-C1 cells synchronized to G1 (**A**), S (**B**) and G2/M (**C**) phases, respectively, by SeMNPV superinfection. Cells were infected with SeMNPV at an MOI of 1. Mock infections were performed by replacing the viral supernatant with the medium.
